# A multi-feature spatial–temporal fusion network for traffic flow prediction

**DOI:** 10.1038/s41598-024-65040-1

**Published:** 2024-06-20

**Authors:** Jiahe Yan, Honghui Li, Dalin Zhang, Yanhui Bai, Yi Xu, Chengshan Han

**Affiliations:** 1 School of Computer and Information Technology, Beijing Jiaotong University, Beijing, 100044 China; 2 School of Software Engineering, Beijing Jiaotong University, Beijing, 100044 China

**Keywords:** Traffic flow prediction, Spatial–temporal data, Transformer, Graph attention network, Engineering, Mathematics and computing

## Abstract

The traffic flow prediction is the key to alleviate traffic congestion, yet very challenging due to the complex influence factors. Currently, the most of deep learning models are designed to dig out the intricate dependency in continuous standardized sequences, which are dependent to high requirements for data continuity and regularized distribution. However, the data discontinuity and irregular distribution are inevitable in the real-world practical application, then we need find a way to utilize the powerful effect of the multi-feature fusion rather than continuous relation in standardized sequences. To this end, we conduct the prediction based on the multiple traffic features reflecting the complex influence factors. Firstly, we propose the ATFEM, an adaptive traffic features extraction mechanism, which can select important influence factors to construct joint temporal features matrix and global spatial features matrix according to the traffic condition. In this way, the feature’s representation ability can be improved. Secondly, we propose the MFSTN, a multi-feature spatial–temporal fusion network, which include the temporal transformer encoder and graph attention network to obtain the latent representation of spatial–temporal features. Especially, we design the scaled spatial–temporal fusion module, which can automatically learn optimal fusion weights, further adapt to inconsistent spatial–temporal dimensions. Finally, the multi-layer perceptron gets the mapping function between these comprehensive features and traffic flow. This method helps to improve the interpretability of the prediction. Experimental results show that the proposed model outperforms a variety of baselines, and it can accurately predict the traffic flow when the data missing rate is high.

## Introduction

With the rapid development of urbanization, the traffic congestion has become one of the most severe problems around the world^1^. To alleviate the traffic congestion, the traffic flow prediction is the fundamental component of the intelligent transportation systems (ITS), which can provide assistants for both travelers and administrators. Then, the travelers can optimize their routes while administrators strengthen traffic managements. Thus, accurate traffic flow prediction is crucial to maintain the stability of the traffic network^2^.

In the field of traffic flow prediction, deep learning is always an effective tool to automatically identify patterns and extract features to analyze the traffic data. Currently, the most of state-of-the-art models regard the traffic flow prediction as a spatial–temporal sequence forecasting task, they capture the spatial–temporal dependencies by integrating different deep networks, such as ASTGCNs^3^, STGNNs^4^, DCRNNs^5^ and GMANs^6^. Although the existing excellent models have achieved satisfactory accuracy, they still ignore some issues. *1) Insufficiency in exploring the traffic influence factors.* The existing models are usually based on the spatial–temporal graphs, they usually emphasize spatial topology relationships, but ignore some important factors, such as the length, width and area of the roads. It is well known that traffic congestions are related to road shapes. Therefore, we should take both road shapes and topology relationships into consideration. Moreover, they don’t explore the importance weights for multiple influence factors. *2) Unable to deal with the discontinuous sequences.* The most of existing models are based on the historical sequence $\left( {X_{t - P + 1} ,X_{t - P + 2} , \ldots ,X_{t} } \right)$ over the past $P$ time slices, and predict the future sequence $\left( {\hat{Y}_{t + 1} ,\hat{Y}_{t + 2} , \ldots ,\hat{Y}_{t + Q} } \right)$ over the next $Q$ time slices. They depend on the continuous sequence with temporal and spatial embeddings to capture the spatial–temporal dependencies. In real-world scenarios, data missing is inevitable due to sensor failures and transmission loss. According to the report of the Texas Transportation Institute, the average data missing rate is about 67%. When the data missing is serious, the data continuity may be disturbed. Obviously, these models cannot obtain the continuous time slices, which may lead to suboptimal performances. *3) The monotonous way for spatial–temporal fusion.* In real life, we can approximately estimate the traffic flow according to our experiences. In this process, we don’t rely on the spatial–temporal sequences, but draw a conclusion based on the multiple features involving weather, holidays, day of week, peak hour and geographical position. Therefore, if we construct the suitable traffic features matrix and learn the mapping function between features matrix and traffic flow, we can provide a new thought for traffic flow prediction. In fact, the disease prediction researches have adopted this way, they judge the disease incidence rate based on multiple features containing age, gender, weight, blood pressure, heart rate, exercise habit and family medical history.

Motivated by the above analysis, this paper proposes a Multi-Feature Spatial–Temporal Fusion Network (MFSTN), whose working principle is just like training a super brain that remember the traffic flow under any condition. Different from existing models that only pay attention to the accuracy, our MFSTN also focuses on the interpretability and feasibility. To improve the representative ability of the multiple traffic features, we propose an Adaptive Traffic Features Extraction Mechanism (ATFEM). In the ATFEM, the complex influence factors have been analyzed to generate the cascaded multiple traffic features, then we assign the importance weights to these features by Lasso regression, finally we extract the Joint Temporal Features Matrix (JTFM) and Global Spatial Features Matrix (GSFM) according to the importance weights and prior knowledge. Especially, the GSFM incorporates the information about road shapes and topology relationships, which makes up the deficiency of existing works and provides a comprehensive approach to portrait the road network. The JTFM contains all the time-varying factors affecting traffic conditions, which is significant to learn the changing trend of the dynamic traffic flow. By fusing these two matrixes, we can obtain the underlying comprehensive spatial–temporal features.

To achieve this goal, the MFSTN is proposed to obtain the underlying comprehensive spatial–temporal features and get the mapping function between these spatial–temporal features and traffic flow. Because the MFSTN predicts the traffic flow based on the feature matrixes instead of the continuous sequences, it is feasible to deploy it in the real scenarios where the data missing rate is high. Our MFSTN consists of the Temporal Transformer Encoder (TTE), Graph Attention Network (GAT), scaled spatial–temporal fusion module and Multi-Layer Perceptron (MLP). Especially, we design the scaled spatial–temporal fusion module, which can solve the problem of inconsistency in temporal and spatial dimensions. It helps to improve the interpretability of the prediction result. For the convenience of reading, we summarize the abbreviations and descriptions as Table 1. The main contributions are summarized as follows.

1. A novel adaptive traffic features extraction mechanism is designed, which extracts the significant traffic features to form the JTFM and GSFM. It is beneficial to improve the representative ability of the traffic features.
2. We propose the MFSTN, which predicts the traffic flow based on the feature matrixes instead of the continuous sequences. Especially, we design a scaled spatial–temporal fusion module to solve the problem of inconsistency in temporal and spatial dimensions. Our MFSTN is beneficial to improve the feasibility when the data missing rate is high, as well as the interpretability for the prediction result.
3. The model is evaluated with the real-world traffic dataset. Experimental results show that the proposed model outperforms a variety of baselines, and it can accurately predict the traffic flow when the data missing rate is high.

**Table 1:** The abbreviations and descriptions.

Abbreviation	Description
MFSTN	Multi-feature spatial–temporal fusion network
ATFEM	Adaptive traffic features extraction mechanism
JTFM	Joint temporal features matrix
GSFM	Global spatial features matrix
TTE	Temporal transformer encoder
GAT	Graph attention network
MLP	Multi-layer perceptron

## Related work

In recent decades, the traffic flow prediction has always been a research hotspot^7^. The early studies construct the traffic simulation systems by leveraging physics parameters and equations, but the theoretical hypothesis must be guaranteed^8,9^. Additionally, the early efforts also attempt to build the time-varying models, such as the autoregressive integrated moving average (ARIMA)^10^, but they are based on the temporal smoothness assumptions. Moreover, the machine learning-based methods, such as k-nearest neighbors (KNN) and support vector regression (SVR)^11^, have the limited data fitting capacity. In summary, the biggest disadvantage of the early methods is that they only consider a single aspect rather than spatial–temporal perspective^12,13^. Benefiting from the development of the deep neural networks (DNNs), the deep learning-based methods regard the traffic flow prediction as a spatial–temporal forecasting task^14,15^. The main idea of the deep learning-based methods is integrating different neural networks to capture the spatial–temporal dependencies from spatial–temporal graphs^16,17^. On the one hand, the recurrent neural networks (RNNs) or temporal convolution networks (TCNs) are usually used to extract the temporal correlations from time slices. On the other hand, the convolutional neural networks (CNNs) or graph neural networks (GNNs) are usually used to extract the spatial correlations from graph relations^18,19^. Besides, the attention mechanisms are more flexible, which can assign importance weights to elements along both the temporal and spatial dimensions^20^. The attention mechanisms can be added to the RNNs-based or CNNs-based model^21,22^, such as DCRNN^5^ or STGCN^4^. In addition, the attention-based models have been proposed, such as ST-WA^23^.

At present, numerous studies have focused on designing deep neural networks by using temporal and spatial attention mechanism^24–26^. For example, Reza et al. conducted a comparative analysis between GRU/LSTM with Transformer, further highlighted the Transformer with the outstanding ability to learn the long-term temporal dependencies^27^. It had been proved that the Transformer can alleviate the vanishing gradients problem of RNNs and overcome the receptive field sizes of TCNs. Huo et al. applied the Transformer to learn the long-term temporal dependencies, while the one-dimensional convolution to learn the short-term temporal dependencies, as well as the graph convolution to capture the spatial dependencies^28^. In addition, more and more researches utilized the parallel computing capability of the Transformer to learn the temporal dependencies between elements regardless of the distance. On the other hand, the graph attention mechanisms can perform well on irregular-structured data. For instance, the GATs can assign different weights to adjacent nodes, which are more applicable to irregular road networks. Guo et al. proposed a novel deep learning traffic forecasting framework named GATCN, which adopted the GAT to capture the spatial dependencies^29^. Huang et al. proposed a multi-relational synchronous graph attention network (MS-GAT), which can learn three embeddings related to channel, temporal and spatial correlations by specific graph attention designs^30^.

Currently, most of the hybrid models have utilized the attention mechanisms to capture the spatial–temporal dependencies in the traffic data. These models have achieved satisfactory accuracy, which can be formulated as $\left\{ {\hat{Y}_{t + 1} ,\hat{Y}_{t + 2} , \ldots ,\hat{Y}_{t + Q} } \right\} = F_{\Theta } \left( {\left\{ {X_{t - P + 1} ,X_{t - P + 2} , \ldots X_{t} } \right\},G} \right)$. We can see that these models depend on the continuous historical sequences to predict the future sequences, which may lead to suboptimal performances when the data missing rate is high. Moreover, these models capture the temporal and spatial dependencies separately, which make original temporal and spatial features are not fused sufficiently. In this paper, we aim to learn the mapping function between features matrix and traffic flow, can be formulated as $\hat{Y}_{i}^{(t)} = F_{\Theta } (X_{G}^{(t)} )$. In this way, we can predict the traffic flow and don’t need to impute the incomplete data when faced with discontinuous data. What’s more, we can conduct the spatial–temporal fusion on the original features.

### Preliminaries

In this section, we collect the multi-source data to analyze the complex traffic influence factors, further extract the multiple traffic features. The main definitions are described below.

*The Traffic Flow Data* records the average travel time of all vehicles on the road at a certain time, which is collected by sensors with abundant time labels, as Table 2. Then, we will extract the temporal features from these time labels, and the definition of temporal features is as follows.

**Table 2:** The traffic flow data.

Road ID	Date	Time interval	Travel time
4377906283422600514	2017-05-06	[2017-05-06 11:04:00,2017-05-06 11:06:00)	3.0
3377906289434510514	2017-05-06	[2017-05-0610:42:00,2017-05-06 10:44:00)	1.0
3377906285934510514	2017-05-06	[2017-05-06 17:46:00,2017-05-06 17:48:00)	35.2

#### Definition 1

(*Temporal Features*): For a time $t$, there are $K$ the temporal features can describe it, such as date, hour, time interval, day of the week, day of the month, peak or off-peak period. Then, the temporal features at time $t$ can be represented as $H^{(t)} = \{ h_{1}^{(t)} ,h_{2}^{(t)} ,...,h_{K}^{(t)} \} \in {\mathbb{R}}^{K}$. Suppose the length of observations is $T$, we can conclude that $H = \left\{ {H^{(t)} |t \in [1,T]} \right\} \in {\mathbb{R}}^{T \times K}$.

The *Road Network Data* records road ID, length, width, area and type, as well as the topology relationships that can be converted to the road network graph, as Table 3. Then, we will extract the spatial features from the road network data. The definition of the road network graph and spatial features are as follows.

**Table 3:** The road network data.

Road ID	Length	Width	Area	Type	Upstream	Downstream
4377906289869500514	57	3	171	1	4377906285525800514	4377906281969500514
9377906285566510514	143	9	1287	1	4377906283759500514#9377906286566510514	4377906282532600514
4377906284525800514	839	3	2517	1	4377906281234600514	4377906280334600514

#### Definition 2

(*Road Network Graph*): The road network topology is an irregular graph can be expressed as $G = (V,E,A)$. $V \in {\mathbb{R}}^{N}$ represents the set of nodes, which reflects road segments. $E \in {\mathbb{R}}^{N \times N}$ represents the set of edges, which reflects the connection relationships of road segments. Moreover, $A \in {\mathbb{R}}^{N \times N}$ is the adjacency matrix, whose values represent the connectivity weights between adjacent road segments.

#### Definition 3

(*Spatial Features*): For a node $v_{i} \in V$ , there are $L$ statistical attributes can be used as spatial features, such as road ID, length, width, area, type, as well as the quantity of upstream and downstream. Then, the spatial features of a node $v_{i}$ can be represented as $S^{(i)} = \{ s_{1}^{(i)} ,s_{2}^{(i)} ,...,s_{L}^{(i)} \} \in {\mathbb{R}}^{L}$. Suppose the number of road segments is $N$, we can conclude that $S = \left\{ {S^{(i)} |i \in [1,N]} \right\} \in {\mathbb{R}}^{N \times L}$.

The *Environment Information Data* includes the holidays, weather and season, as Table 4. We mark the holidays date with 1, and the non-holidays date with 0. As for weather, we collect the maximum temperature, minimum temperature, wind force, wind direction, precipitation condition. Moreover, we mark the four seasons respectively. Then, we will extract the external features from the environment information data. The definition of the external features is as follows.

**Table 4:** The environmental data.

Date	Min/max temperature	Wind direction	Wind force	Rainfall	Holidays	Season
2017-05-01	18 ℃/27 ℃	1.5π	3	1	1	Spring
2017-05-06	19 ℃/28 ℃	1.75π	3	0	0	Spring
2017-05-07	16 ℃/28 ℃	1.75π	3	1	0	Spring

#### Definition 4

(*External Features*): We know that the traffic flow data can easily be affected by external factors, such as holiday, season and weather. Then, we denote the external features of a day $d$ as $F^{(d)} = \{ f_{1}^{(d)} ,f_{2}^{(d)} ,...,f_{Z}^{(d)} \} \in {\mathbb{R}}^{Z}$. Suppose the days of observations is $D$, we can conclude that $F = \left\{ {F^{(d)} |d \in [1,D]} \right\} \in {\mathbb{R}}^{D \times Z}$.

#### Definition 5

(*Traffic Flow Prediction*): The task of traffic flow prediction is to learn a mapping function $F_{\Theta }$ that maps the traffic features matrix $X_{G}^{(t)}$ to the traffic flow $\hat{Y}_{i}^{(t)}$, as follows.

1 $$\hat{Y}_{i}^{(t)} = F_{\Theta } \left( {X_{G}^{(t)} } \right),$$

where the traffic features matrix is $X_{G}^{(t)} = \{ H^{(t)} ,S^{(i)} ,F^{(d)} \}$. After learning the mapping function $F_{\Theta }$, we can predict the traffic flow at future time.

## Methodology

**Figure 1:**
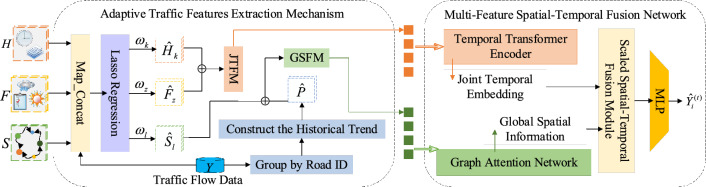
The overall architecture of our method.

Although we have extracted the multiple traffic features that play a decisive role in affecting the traffic condition, at present, these features are still shallow, discrete and coarse. The idea of our proposed method is to employ the latent united representation of spatial–temporal features. The pipeline of our method is shown in Fig. 1, which consists of the following two new proposed constituent parts. Firstly, we design the ATFEM, an adaptive traffic features extraction mechanism, which selects significant features by Lasso regression. Consequently, we select the important temporal features, spatial features and external features. Then, we combine all the time-varying features to form JTFM, including the selected temporal features and external features. And we portrait the road network by uniting the selected spatial features and historical statistics to form GSFM. The JTFM can serve as the unique temporal symbol to indicate temporal traffic condition. Meanwhile, the GSFM reflects the global road network states by considering features of the road shapes, topological relationships and historical trends. In this way, we enhance the representative ability of multiple traffic features. Secondly, we propose the MFSTN, a multi-feature spatial–temporal fusion network, which consists of TTE, GAT, our scaled spatial–temporal fusion module and MLP. Based on above components, the MFSTN has the capacity to dig out the implicit correlations in two feature matrixes and fuse the latent representation of spatial–temporal features, finally get the mapping function between these features and traffic flow. Next, we will introduce the ATFEM and MFSTN in detailed.

### The adaptive traffic features extraction mechanism

The traffic flow data $X \in {\mathbb{R}}^{Q \times C}$ records the average travel time of all vehicles on the road at a certain time. The $Q$ is the number of records and $C$ is the number of original columns. Because there are a lot of missing data, then $Q \ll NT$. Firstly, we split the time labels to form the temporal features, then $X \in {\mathbb{R}}^{Q \times (K + C)}$. Secondly, we concatenate the $X$ and $F$ by date, then $X \in {\mathbb{R}}^{Q \times (K + Z + C)}$. Thirdly, we concatenate the $X$ and $S$ by road ID, then $X \in {\mathbb{R}}^{Q \times (K + L + Z + C)}$. Finally, we delete the columns with repetitive meanings, then $X \in {\mathbb{R}}^{Q \times (K + L + Z + 1)}$ that include the temporal features, spatial features, external features and traffic flow. Next, we adopt the Lasso regression to explore the importance weights of multiple traffic features, which is as

2 $$y = \omega_{0} + \omega_{1} x_{1} + \omega_{2} x_{2} + \cdots + \omega_{m} x_{m} + b,$$

3 $$\underbrace {{\left[ {\begin{array}{*{20}c} {y_{1} } \\ {y_{2} } \\ \vdots \\ {y_{n} } \\ \end{array} } \right]}}_{y} = \underbrace {{\left[ {\begin{array}{*{20}c} 1 & {x_{11} } & {x_{12} } & \cdots \\ 1 & {x_{21} } & {x_{22} } & \cdots \\ \vdots & \vdots & \vdots & \cdots \\ 1 & {x_{n1} } & {x_{n2} } & \cdots \\ \end{array} \quad \begin{array}{*{20}c} {x_{1m} } \\ {x_{2m} } \\ \vdots \\ {x_{nm} } \\ \end{array} } \right]}}_{x}\;\underbrace {{\left[ {\begin{array}{*{20}c} {\omega_{0} } \\ {\omega_{1} } \\ \vdots \\ {\omega_{m} } \\ \end{array} } \right]}}_{\omega } + \underbrace {{\left[ {\begin{array}{*{20}c} {b_{0} } \\ {b_{1} } \\ \vdots \\ {b_{m} } \\ \end{array} } \right]}}_{b},$$

where $y$ represents the traffic flow and $\omega$ is the importance weights. We select the significant features with high importance weights and make the $\hat{H}$ ,$\hat{S}$ ,$\hat{F}$ to represent the selected temporal features, spatial features, external features respectively. Then, we adopt statistics to construct the historical trends of each road, which can reflect the historical road network states. Since the traffic flow at night is free flow, we select the traffic flow at 6:00 am to 23:00 pm to construct the historical trends. We use the $p_{h}^{d} (i)$ to represent the traffic trend of road $i$ at hour $h$ on day $d$, which is as

4 $$p_{h}^{d} (i) = \frac{{\sum\nolimits_{\tau = 1}^{30} {y_{h,\tau }^{d} (i)} }}{30},$$

where $\tau$ is the number of time slices. Because we collect the traffic flow data in two minutes, there should be 30 time slices in an hour. Then we use $p_{h} (i)$ to represent the average traffic trend of road $i$ of the hour $h$ in $D$ days, which is as

5 $$p_{h} (i) = \frac{{\sum\nolimits_{d = 1}^{D} {p_{h}^{d} (i)} }}{D}.$$

Then, we have $\hat{P} = \{ p_{h} (i)|h \in [6,23],i \in [1,N]\}$ to reflect the historical trends of the road network. Finally, we combine the $\hat{H}$ and $\hat{F}$ to form the JTFM, which contains all the time-varying factors that are significant to learn the changing trend of the dynamic traffic flow. And we combine the $\hat{S}$ and $\hat{P}$ to form the GSFM that incorporates the information about road shapes, topology relationships and historical trends, which can reflect the global road network states.

The principle of the ATFEM is automatically selecting important features that play a decisive role in affecting the traffic condition. Its adaptability is reflected in adapting to different traffic condition or data characteristics. On the one hand, we consider the multiple traffic features as trainable variables that affect the traffic condition. The ATFEM can automatically learn the relationships between features and targets, no matter how those relationships change. On the other hand, our method is not limited by the number of features, which can be applied to different data characteristics.

### The multi-feature spatial–temporal fusion network

The MFSTN consists of TTE, GAT, our scaled spatial–temporal fusion module and MLP, where the TTE is used to get the joint temporal embedding and the GAT is utilized to aggregate the global spatial information. Then, our scaled spatial–temporal fusion module can automatically learn optimal fusion strategy to get the latent united representation of spatial–temporal features. Finally, the MLP outputs the prediction result. Next, we will introduce the key components.

**Figure 2:**
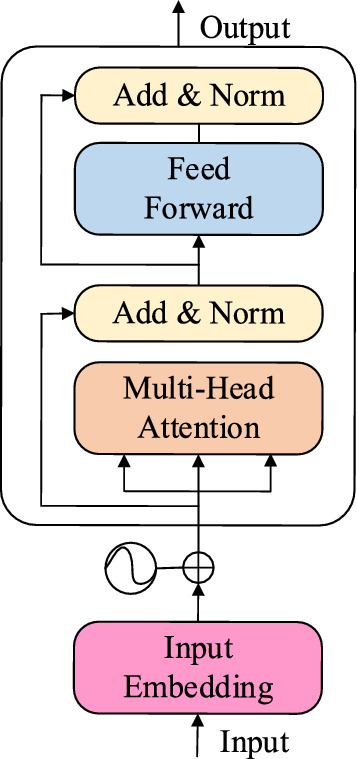
The framework of TTE.

*Temporal Transformer Encoder* For the temporal features, we have obtained the JTFM, which contains all the time-varying factors that are significant to learn the dynamic trend. Then, we will use the TTE to encode JTFM to get the joint temporal embedding, which can serve as the unique temporal symbol to distinguish dynamic traffic condition. As Fig. 2 shown, the encoder is constituted by the multi-head attention mechanism, the feed forward layer, and the add & norm layer.

The self-attention mechanism can be realized by (6), whose internal logic is as

6 $$attention\,\left( {Q,K,V} \right) = soft\max \left( {\frac{{QK^{T} }}{{\sqrt {d_{k} } }}} \right)V,$$

where $d_{k}$ is the input embedding dimension, and the input matrix $J \in {\mathbb{R}}^{{Q \times d_{k} }}$ is the JTFM after dimension embedding. And matrix of $Q$, $K$, $V$ can be computed by the input matrix $J$ and linear transformation matrix of $W^{Q}$, $W^{K}$,$W^{V} \in {\mathbb{R}}^{{d_{k} \times q}}$, $q$ is the linear mapping dimension. Then, we can get the value of $Q$, $K$, $V \in {\mathbb{R}}^{Q \times q}$ by formula of $Q = JW^{Q}$, $K = JW^{K}$, $V = JW^{V}$.

The multi-head attention mechanism contains $M$ self-attention mechanisms, whose formula is as

7 $$multihead( \cdot ) = concat\left( {attention_{1} ( \cdot ),...,attention_{M} ( \cdot )} \right)W^{o} ,$$

where $W^{o} \in {\mathbb{R}}^{{(q \times M) \times d_{k} }}$ is the output parameter. Moreover, the Transformer appends an add & norm layer after the multi-head attention, which means the output of the multi-head attention should add with the input, then pass a normalization layer. In this way, we can integrate the original input and the intermediate output to better mining data. The process can be shown as (8)

8 $$add\,\&\, norm = layernorm\left( {J + multihead\left( J \right)} \right).$$

After that is the feed forward layer that contains two fully-connected layers, whose formula is as

9 $$feedforward = \max \left( {0,\;Res^{\prime } W_{1} + b_{1} } \right)W_{2} + b_{2} ,$$

in which ${\text{Re}} s^{\prime }$ is the intermediate output of the add & norm layer, and $W_{1}$, $W_{2}$, $b_{1}$, $b_{2}$ are the corresponding learnable parameters. Then the feed forward layer also followed by an add & norm layer, as (10) shown

10 $$add\,\&\, norm = layernorm\,\left( {{\text{Re}} s^{\prime } + feedforward\left( {{\text{Re}} s^{\prime } } \right)} \right).$$

As we known, the Transformer has the outstanding performance in the machine translation tasks, it’s encoder also has powerful ability to encode the temporal factors. Based on the TTE, we can get joint temporal embedding. Moreover, the multi-head attention mechanism can dig out the hidden correlation between the temporal factors, so it can provide the similar embedding for similar temporal condition. Finally, the TTE outputs the joint temporal embedding $\hat{M}_{T} (\theta ) \in {\mathbb{R}}^{{Q \times c_{t} }}$, where the $Q$ is the number of records in traffic flow data, and $c_{t}$ represents temporal embedding dimension.

*Graph Attention Network* For the spatial features, we have obtained the GSFM, which incorporates road shapes, topological relationships and historical trends. As we known, if two roads are adjacent in space or similar in shape, they may exhibit a certain homogeneity. Moreover, the city can be divided into different functional areas, such as business areas and residential areas. If two roads are in the same functional area, their traffic flows show the similarity. However, this kind of similarity is implicit and not easy to measure. Fortunately, the GAT can compute the similarity of adjacent roads based on the graph attention mechanism, then assign different weights according to neighboring similarity, finally aggregate the global spatial features as the new representation. Therefore, we adopt the GAT to aggregate spatial features to get the global spatial representation. The input of the GAT is the GSFM multiplied by $A$, where $A \in {\mathbb{R}}^{N \times N}$ is the adjacency matrix and the GSFM provide the GAT with global road network states. Then the GAT can compute the roads similarity from the comprehensive perspective.

**Figure 3:**
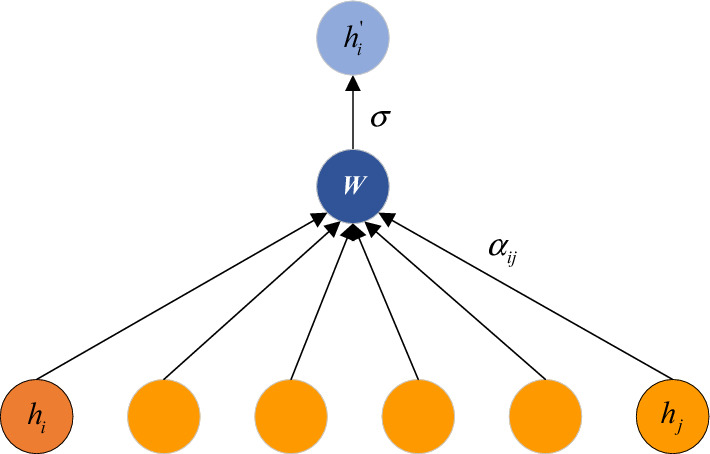
The features aggregation of GAT.

As Fig. 3 shown, the GAT can assign different attention coefficients to all neighbor nodes, then compute a weighted average of the spatial features of all neighbor nodes as the new representation for node $v_{i}$. The calculation formula for new status of node $v_{i}$ is as follows

11 $$h_{i}^{\prime } = \sigma \left( {\sum\limits_{{j \in {\rm N}_{i} }} {\alpha_{ij} } \cdot Wh_{j} } \right),$$

in which $\sigma$ represents the nonlinear activation function, and $\alpha_{ij}$ are used to normalize across all neighbors $v_{j} \in {\rm N}_{i}$ using softmax function:

12 $$\begin{aligned} \alpha_{ij} & = {\text{softmax}}_{j} \left( {e(h_{i} ,h_{j} )} \right) \\ & = \frac{{\exp \left( {e(h_{i} ,h_{j} )} \right)}}{{\sum\nolimits_{{j^{\prime } \in {\rm N}_{i} }} {\exp \left( {e(h_{i} ,h_{{j^{\prime } }} )} \right)} }}, \\ \end{aligned}$$

where $e(h_{i} ,h_{j} )$ measures the similarity between the node $v_{i}$ and node $v_{j}$, and it is defined as:

13 $$e\left( {h_{i} ,h_{j} } \right) = {\text{LeakyReLU}}\left( {{\text{a}}^{T} \cdot \left[ {{\text{Wh}}_{i} {\text{||Wh}}_{j} } \right]} \right){.}$$

And $a$,$W$ are learning parameters, $\parallel$ represents vector concatenation. The definition of GAT has been shown from formula (11) to (13). Finally, the GAT outputs the global spatial information $\hat{M}_{G} (\theta ) \in {\mathbb{R}}^{{N \times c_{g} }}$, where the $N$ is the number of roads, and $c_{g}$ is the output dimension.

*Scaled Spatial–Temporal Fusion Module* To effectively fuse the $\hat{M}_{T} (\theta )$ and $\hat{M}_{G} (\theta )$, we propose a scaled spatial–temporal fusion module. Firstly, we will scale dimensions of the $\hat{M}_{T} (\theta )$ and $\hat{M}_{G} (\theta )$ to concatenate them to get the comprehensive representation of the spatial–temporal features. It is worth mentioning that we keep the road ID in the input of TTE, but it does not participate in the training process. Therefore, we could concatenate them by road ID, the process is as follows

14 $$\hat{M}_{T} \parallel \hat{M}_{G} = \left\{ {\hat{m}_{t} \parallel \hat{m}_{g} |\hat{m}_{t} \left[ {road.id} \right] = \hat{m}_{g} \left[ {road.id} \right]} \right\},$$

where $\hat{m}_{t} \in \hat{M}_{T}$, $\hat{m}_{g} \in \hat{M}_{G}$ and $\hat{M}_{T} \parallel \hat{M}_{G} \in {\mathbb{R}}^{{Q \times (c_{t} + c_{g} )}}$. Then, we use a gating unit to automatically learn optimal fusion weights for different features, then the latent united representation of spatial–temporal features ${\mathcal{R}}$ is as

15 $${\mathcal{R}}\left( {0:c_{t} - 1,\;:,} \right) = {\mathcal{H}} \odot \hat{M}_{T} (\theta ),{\mathcal{R}}\left( {\;:,c_{t} :\,c_{t} + c_{g} } \right) = {\mathcal{H}} \odot \hat{M}_{G} (\theta ),$$

where ${\mathcal{H}}$ is the gating unit that used to find optimal fusion parameters for temporal and spatial features, which is pretrained by linear layers as

16 $${\mathcal{H}} = \delta \left( {W_{T} \hat{M}_{T} (\theta ) + W_{G} \hat{M}_{G} (\theta )} \right),$$

where $\delta$ is the activation function, the $W_{T}$ and $W_{G}$ are trainable parameters that use to balance the temporal and spatial features.

*Multi-Layer Perceptron* Finally, we use the MLP to output the prediction result, which is as

17 $$\hat{Y} = MLP({\mathcal{R}}).$$

In this way, we predict the traffic flow based on the latent united representation of spatial–temporal features, which has the advantage to fully fuse the temporal and spatial features.

### Experiment

In this section, we conduct experiments on the real-world dataset to evaluate the performance of our method. First, we describe the experimental dataset and data preprocessing. Then, we introduce significant features selected by our ATFEM, further show the effectiveness of our ATFEM. Next, we introduce the baselines, evaluation metrics and experiment settings to present the prediction accuracy of our MFSTN. Finally, we verify the feasibility of the method through an application case.

### Description and data preprocessing

We conduct experiments based on the real-world dataset from Guizhou, China. This dataset includes the traffic flow data and road network data, and we collect the environment information data as the auxiliary.

*Traffic flow data* is collected from 2017/03/01 to 2017/07/01, and includes 98 days of workday, 15 days of weekend, 9 days of holiday. The time interval is two minutes, there are over 6 million records for 132 roads.

*Road network data* depicts the attributes of 132 roads. It is important to note that there is a great difference in road shapes, where the ${\text{length}} \in [5,839]$,${\text{width}} \in [3,15]$, and ${\text{area}} \in [48,5010]$, as well as quantity of upstream and downstream is 0 ~ 4.

*Environment information data* is collected from 2017/03/01 to 2017/07/01, which includes the characteristics of holiday, season and weather.

**Figure 4:**
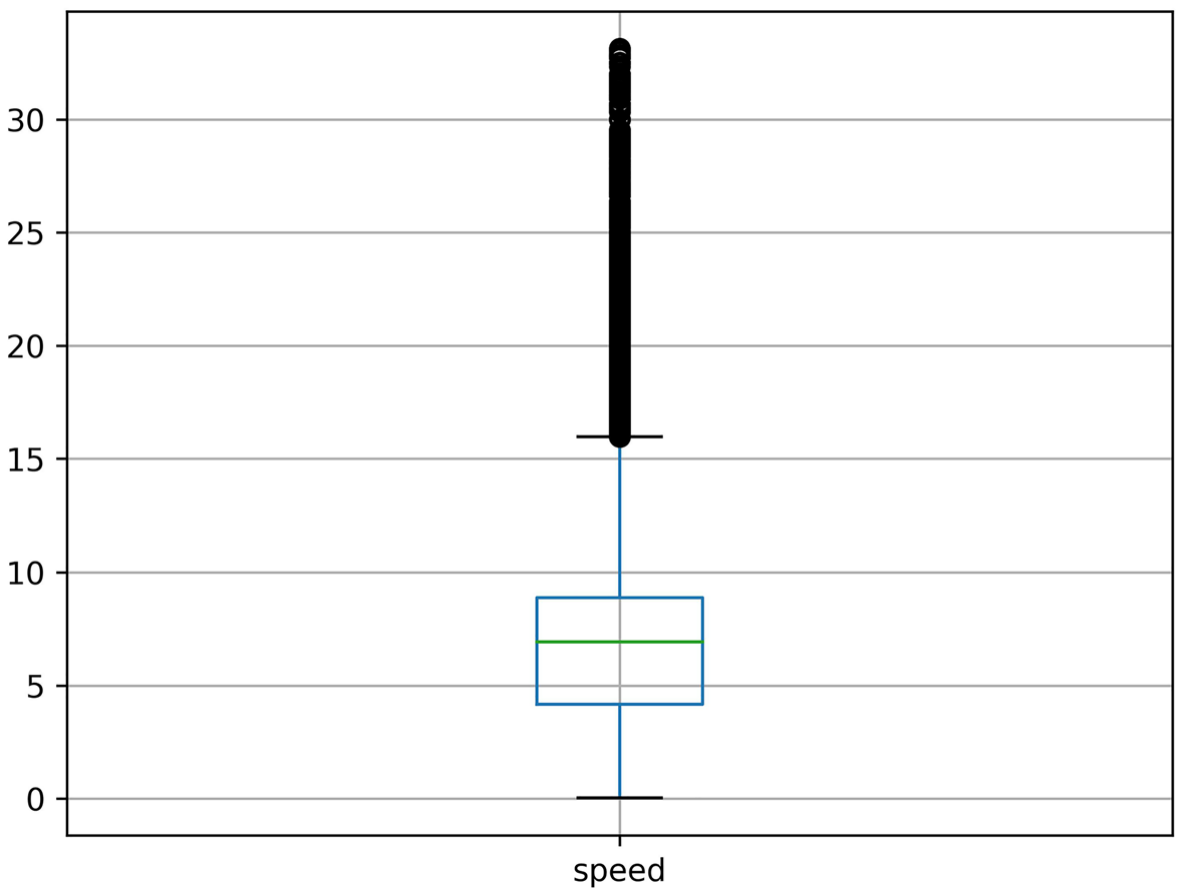
The outlier situation.

**Figure 5:**
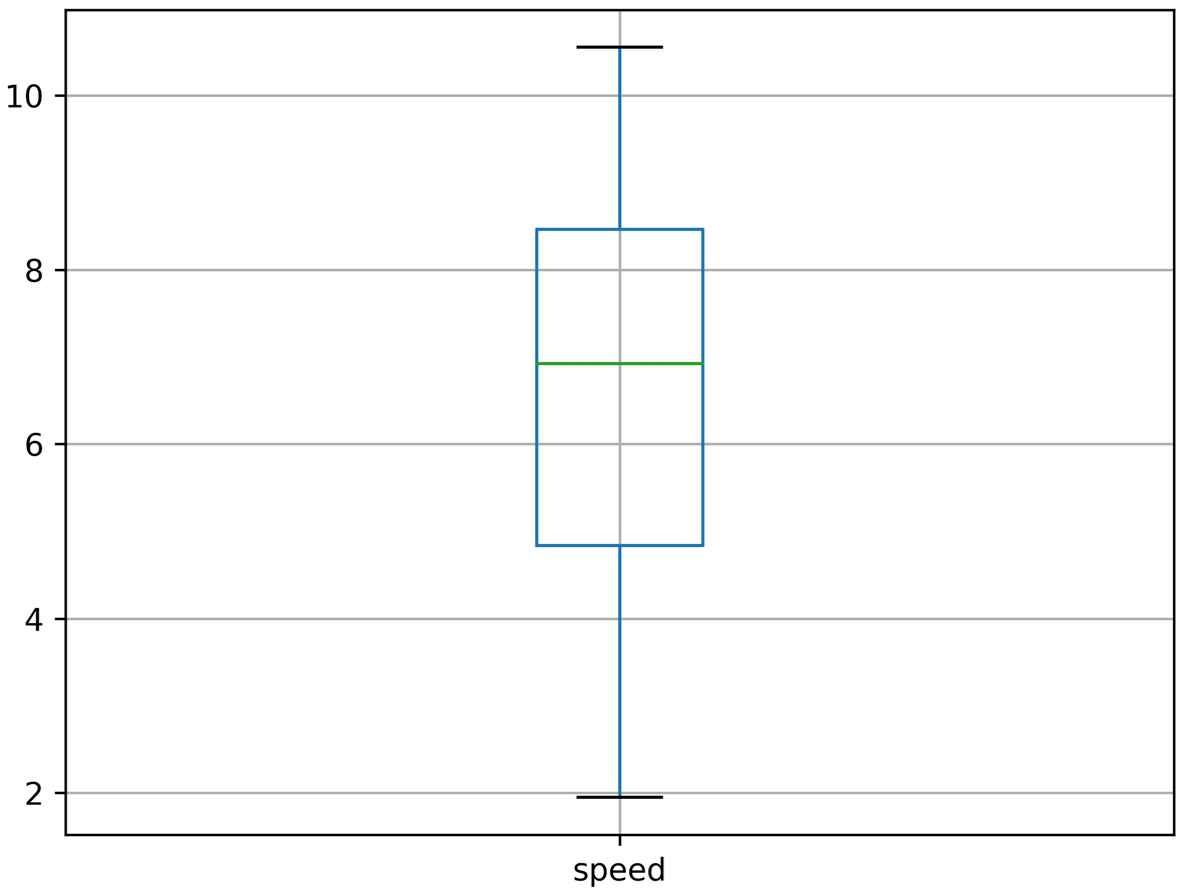
The outlier deletion.

**Figure 6:**
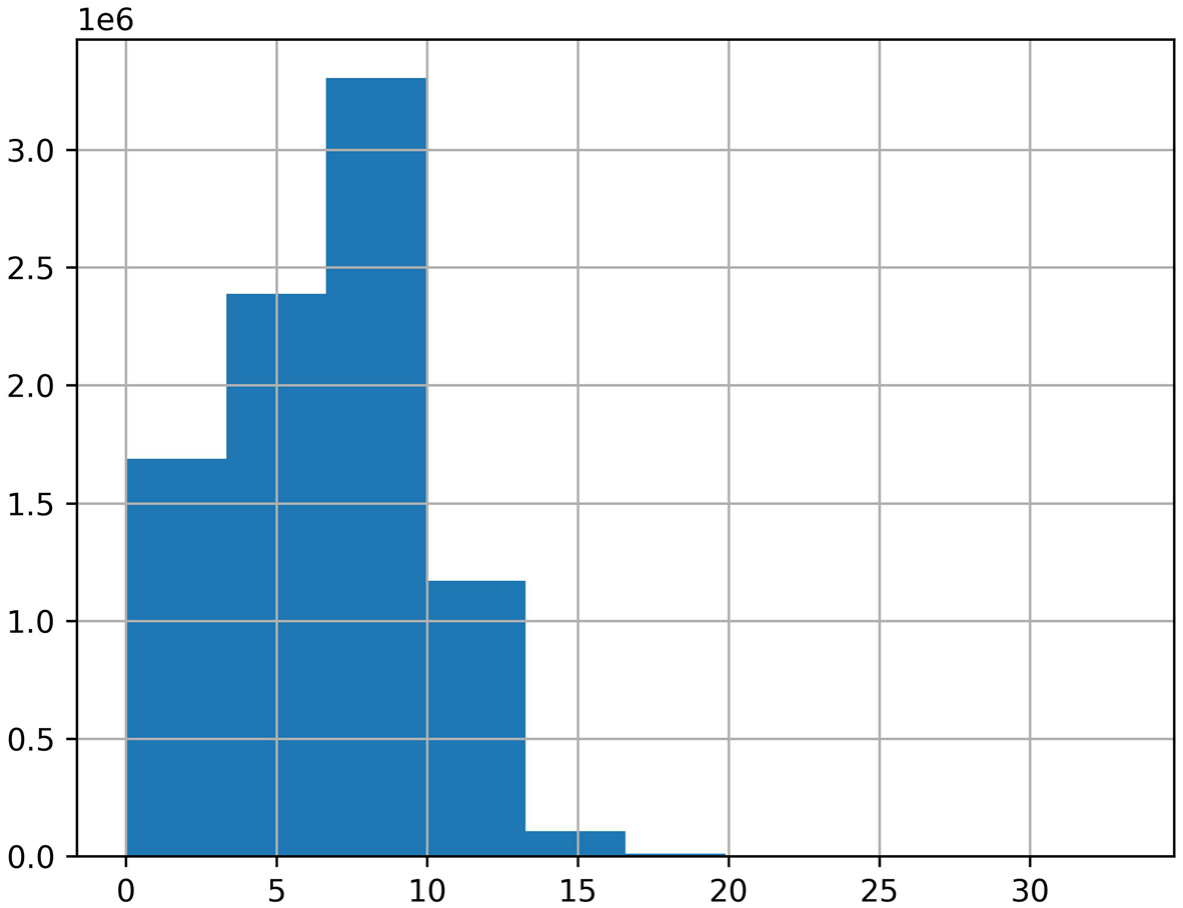
The long-tail distribution.

**Figure 7:**
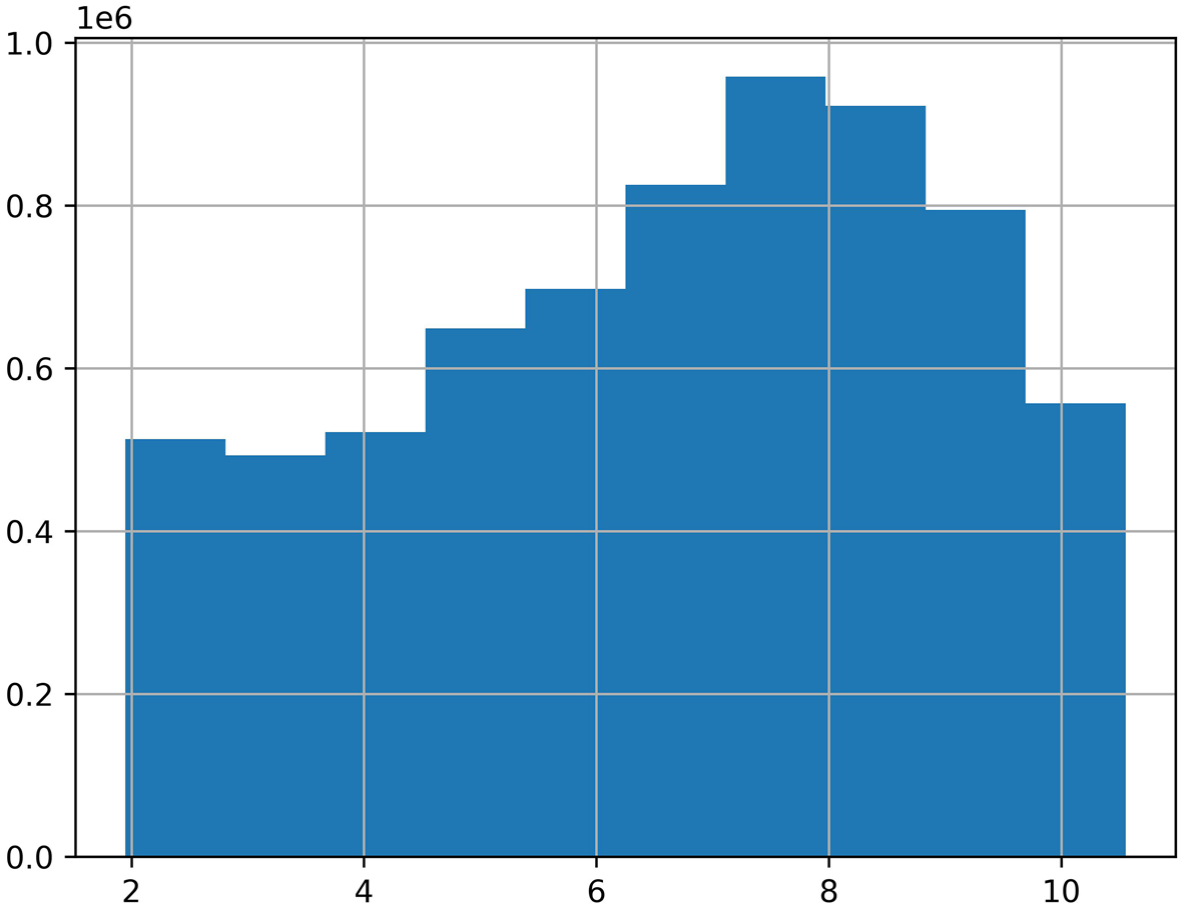
The transformation distribution.

Since the dataset is collected from the real scenario, there are a lot of missing values, abnormal values, and error values. The data missing situation is serious. Half the roads have the data missing rate of more than 20%. Some roads have the data missing rate as high as 50% to 90%. The serious data missing situation has disturbed the data continuity, which leads the spatial–temporal sequences-based methods are not applicable. Our spatial–temporal features-based method don’t need to impute the incomplete data, which reduces the complexity of processing missing values. For the abnormal values, we cannot directly categorize all outliers as error values. Because the average travel time is related to the road length, some abnormal outliers may reflect the longer roads. Then, we can’t delete all the outliers as error values. What's more, we judge traffic congestion based on the average travel time, but it may reflect traffic congestion or longer roads. This phenomenon may cause some confusion. Therefore, we temporarily transform the average travel time to the average speed, so the difference between road length can be eliminated. Then, the distribution of the average speed can be shown as Fig. 4. We can see that there are still many outliers in Fig. 4, then we just delete them as Fig. 5. In addition, the data distribution is a long-tail distribution as Fig. 6, then we need conduct the log transformation. The transformation can be shown as Fig. 7, we could see that the data is more well-distributed. After that, we transform the average speed back to the average travel time.

For the traffic flow data, we split its time labels to form the temporal features. For example, we split the [2017–05-06 11:04:00, 2017-05-06 11:06:00) to “2017”, “05–06”, “11:04:00”, “11:06:00”, then we delete the column of year, because its variance is zero and doesn’t have the ability to differentiate records. In addition, we append some features, such as “day of the week”, “day of the month”, “peak or off-peak period”. These temporal features can not only uniquely identify a record, but also reflect some periodicities, such as the daily periodicity, weekly periodicity, monthly periodicity. For the environment information data, which includes the external features, such as “date”, “holidays”, “season”, “rainfall”, “maximum temperature”, “minimum temperature”, “wind force”, “wind direction”. We concatenate the traffic flow data and environment information data according to the date, then we get all the time-varying features. For the road network data, we combine the spatial features, such as “road ID”, “length”, “width”, “area”, “type”, “upstream quantity”, “downstream quantity”. We concatenate the traffic flow data and road network data according to the road ID, then we get all the spatial–temporal features. Next, we will explore the importance weights between these features by Lasso, further select the significant features can play an important role in affecting traffic condition. The result can be shown as Table 5. In Table 5, we divide the importance degree into four groups according to the normalized importance weights $\overline{\omega }_{ * }$, as follows

18 $$\left\{ \begin{gathered} {\text{High - level}}:\;\;\overline{\omega }_{ * } \in [0.3,1] \hfill \\ {\text{Medium - level}}:\;\;\overline{\omega }_{ * } \in [0.1,0.3) \hfill \\ {\text{Low - level}}:\;\;\overline{\omega }_{ * } \in [0.001,0.1) \hfill \\ {\text{Unrelated}}:\;\;\overline{\omega }_{ * } \in [0,0.001) \hfill \\ \end{gathered} \right..$$

**Table 5:** The importance of multiple features.

Importance	Features
High-level	“length”, “width”, “area”, “time interval”, “day of the week”, “peak period”
Medium-level	“rainfall”, “holidays”, “upstream quantity”, “downstream quantity”
Low-level	“season”, “month”, “wind direction”
Unrelated	“type”, “day of the month”, “wind force”, “maximum temperature”, “minimum temperature”

Specially, the “date” and “road ID” are the indispensable identifiers that beyond the limitation of importance weights. We can see that the features related to the road shapes have high importance, while the features related to the topology relationships have medium importance. That means our consideration of the road shapes has important implications. Moreover, we find that the feature of “rainfall” has a little bit bigger weight than the “holidays”. We attribute this phenomenon to the randomness of rainfall leads to the greater ability to differentiate. Another important finding is that the “wind direction” has the low importance, not like the others external features are unrelated. This is because that the “wind direction” is related to “season”, and the “season” can affect the travel habits. Through analysis, we conclude that the south wind predominates here in summer. This shows that our method can dig out the hidden factors. Next, we will demonstrate the effect of our model on these features.

### Evaluation metrics

Five metrics are employed to measure the performance of our model, namely, Mean Absolute Error (MAE), Mean Square Error (MSE), Root Mean Squared Error (RMSE), Coefficient of Determination (R^2^) and Mean Absolute Percentage Error (MAPE), which are defined as (19) to (23).

19 $$MAE = \frac{1}{\left| Q \right|}\sum\limits_{i \in Q} {\left| {y_{i} - \hat{y}_{i} } \right|}$$

20 $$MSE = \frac{1}{\left| Q \right|}\sum\limits_{i \in Q} {\left( {y_{i} - \hat{y}_{i} } \right)^{2} }$$

21 $$RMSE = \sqrt {\frac{1}{\left| Q \right|}\sum\limits_{i \in Q} {\left( {y_{i} - \hat{y}_{i} } \right)^{2} } }$$

22 $$R^{2} = 1 - \frac{{\sum\nolimits_{i = 1}^{Q} {\left( {y_{i} - \hat{y}_{i} } \right)^{2} } }}{{\sum\nolimits_{i = 1}^{Q} {\left( {y_{i} - \overline{y}_{i} } \right)^{2} } }}$$

23 $$MAPE = \frac{1}{\left| Q \right|}\sum\limits_{i \in Q} {\left| {\frac{{y_{i} - \hat{y}_{i} }}{{y_{i} }}} \right|}$$

The $y_{i}$ represents the ground truth, $\hat{y}_{i}$ denotes the predicted value, $\overline{y}_{i}$ is the average value computed by $\overline{y}_{i} = \tfrac{1}{\left| Q \right|}\sum\nolimits_{i = 1}^{Q} {y_{i} }$. And $Q$ is the number of observed records.

### Baselines

As we known, most of existing models are based on the continuous spatial–temporal sequences, which are not applicable to the scenarios with high data missing rate. To this end, we propose the spatial–temporal features-based method. Therefore, our model is not comparable to most of existing models. To prove the effectiveness of our method, five baselines are employed for the experimental comparisons. The Historical Average (HA) is a representative statistical analysis method and the Support Vector Regression (SVR) is a typical machine learning method, both are usually compared to the existing models. To verify the advantage of the spatial–temporal features extraction, the Fully Connected Network (FCN) conducts the prediction without deeply process features. Besides, the Transformer also acts as the baseline to show the importance of global spatial features. Moreover, the LSTM-GAT combines the long short-term memory and graph attention network is used to show the advantage of our joint temporal embedding.

### Experimental results

As for the hyperparameter settings, we train our model using the Adam optimizer for 100 epochs with a batch size of 12,000 and an initial learning rate of 5e-5. The layers of TTE and GAT is set to 2 and the number of attention heads is 2. The layers of MLP is set to 5. The $c_{t} = 5$, $c_{g} = 5$.We split the dataset with ratio 7: 3 into training set and test set by the time. As the Table 6, we can see that our MFSTN gets the best prediction performance by considering five evaluation metrics. These five metrics have different meanings, the smaller value of the MAE, MSE, RMSE, MAPE means the better prediction result, while the greater value of the R^2^ means the better prediction result. Since the MSE computes the square of the prediction error, its value is much greater than others.

**Table 6:** The prediction comparison on Guizhou dataset.

Method	MAE	MSE	RMSE	R2	MAPE
HA	4.2939	25.8327	5.0826	0.0801	18.1406
SVR	2.9184	11.5318	3.3959	0.1328	12.3207
FCN	3.0079	13.2737	3.6433	0.1258	12.7584
Transformer	2.7888	9.8345	3.1360	0.1878	11.9192
LSTM-GAT	2.9153	12.0708	3.4743	0.1339	12.7275
MFSTN	**2.2639**	**8.0175**	**2.8315**	**0.2121**	**9.1711**

Significant values are in bold.

As Table 6, the HA has the lowest prediction accuracy, because it only relies on the historical average to make a prediction and doesn’t consider the spatial–temporal features that affect the traffic condition. However, the prediction result is not particularly bad, which shows the historical average can reflect the general trend of traffic patterns. This can be further proved that the ATFEM adds the historical trend into features matrix is effective. The fourth is the SVR and the fifth is the FCN. The SVR fits the relationship between variables to output the prediction result. The FCN memories the multiple features by deep network structure and maps these features to the output. We can find that the SVR has better prediction accuracy than the FCN. That shows the importance of exploring the relationships between features, further proves that appropriate feature engineering is more effective than increasing model depth. Compared to our MFSTN, the LSTM-GAT adopts the LSTM to obtain the temporal embedding. However, the prediction accuracy of the LSTM-GAT is worse than the Transformer. This demonstrates that time-varying features play a major determining role. Moreover, the Transformer has the outstanding performance to deal with temporal features. But the prediction accuracy of the Transformer is not better than the MFSTN. That shows the global spatial information aggregated by GAT can improve the prediction performance.

From the Table 6 we doubt whether the prediction error can be further optimized or not. But the biggest obstacle is the data quality, the experimental dataset comes from the real-scenario, there are a lot of missing data, and we cannot get the real value of the missing data. It is worth mentioning that the data missing rate of two roads is more than 90%, which will affect the total prediction performance. As we known, the data quality determines the upper limit of artificial intelligence models, and models only infinitely approach to this upper limit. Although affected by data quality, our model still achieves the satisfactory results.

**Figure 8:**
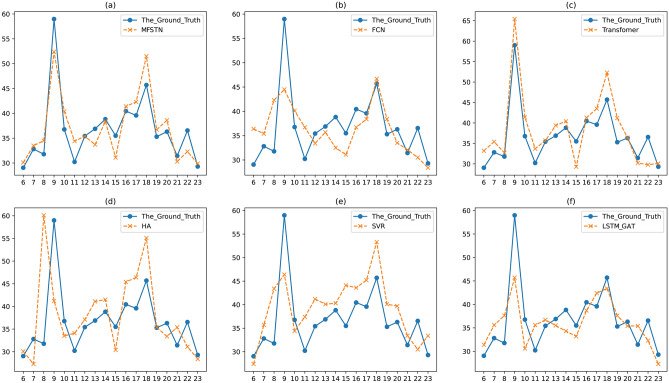
The prediction result of a road on June 1st.

**Figure 9:**
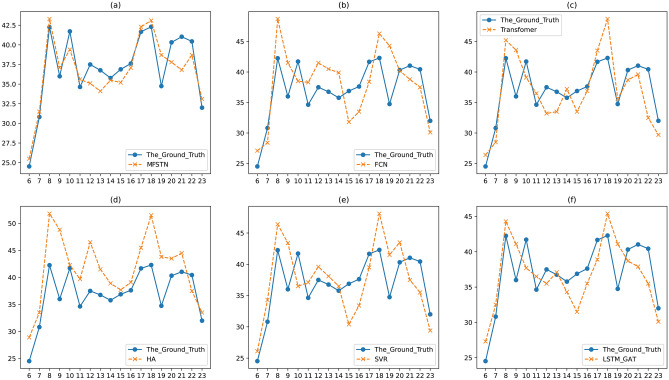
The prediction result of a road on June 10th.

**Figure 10:**
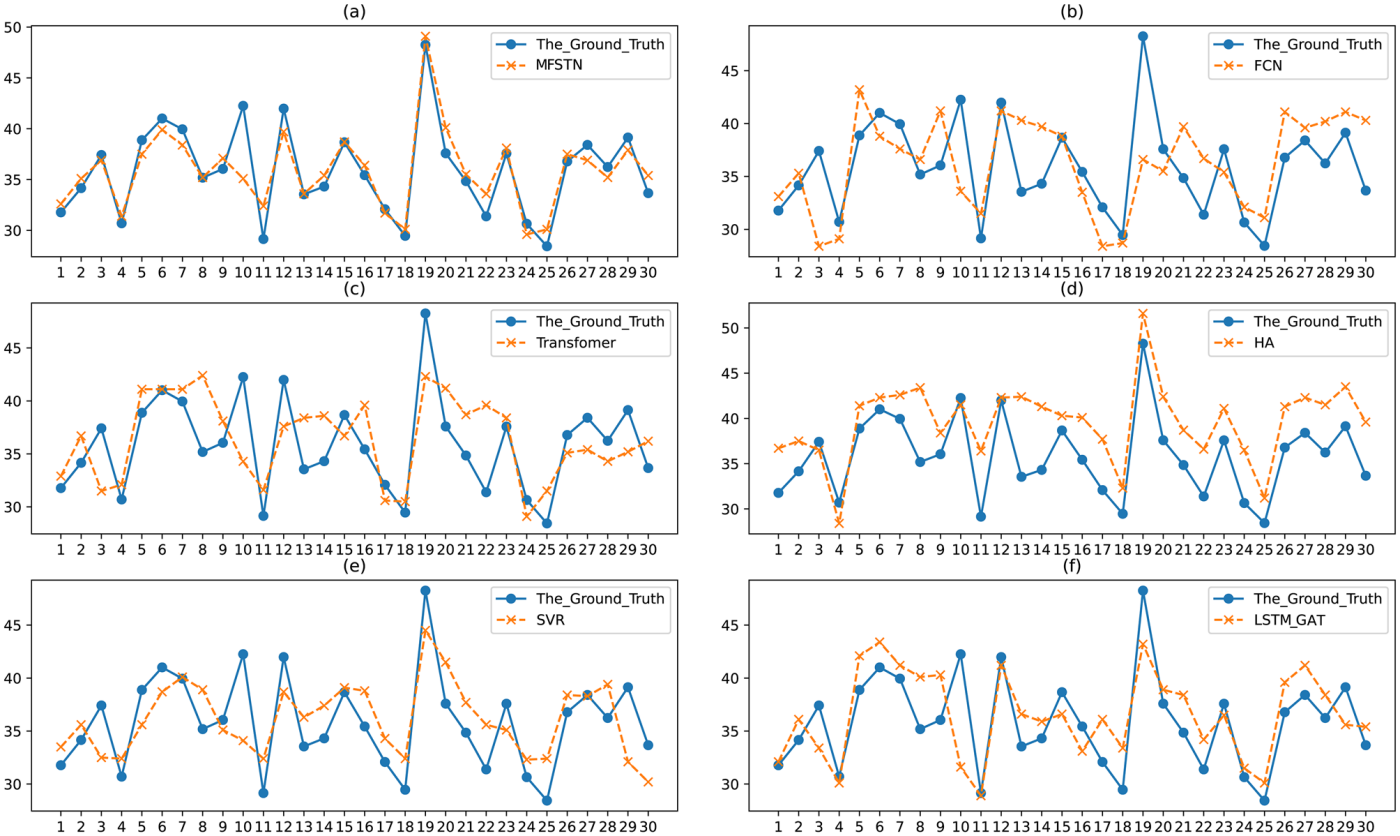
The prediction result of a road from June 1st to 30th.

Next, we give a case to show the comparison between the ground truth and the prediction result. Figure 8 shows the comparison of a road on June 1st that is a workday, while Fig. 9 shows the comparison on June 10th that is a weekend. In the Fig. 8 and Fig. 9, we list the prediction result and the ground truth from 6:00 am to 23:00 pm. Since the night traffic flow is in a free state, it’s not significant to predict the traffic flow at 0:00 am to 5:00 am. From Fig. 8a–f, we can see that the proposed MFSTN model has the best prediction result on workdays. In Fig. 8a, we can observe that the predicted value is consistent with the real value, with only a few points fluctuate. Moreover, the MFSTN model can accurately predict the travel time at morning peak and evening peak, while other baselines cannot. We can observe that the morning peak occurs around 9:00 am, while the evening peak occurs around 6:00 pm. From Fig. 9a–f, our MFSTN model can also achieve the best prediction result on weekends. But there is no obvious morning peak and evening peak in Fig. 9. Because the traffic condition on weekends is different from the workdays, people usually prefer more casual travel patterns on weekends. That shows that our model can accurately capture the traffic patterns both on workdays and weekends. Then, we will show the long-term prediction accuracy of the proposed MFSTN. Figure 10 illustrates the comparison between the ground truth and the prediction result of a road in a month. The prediction range is from June 1st to 30th, the prediction interval is the 8:00 am. Compared with the baselines, we can see that the MFSTN model achieves the best prediction result within a month. That shows our model has stable prediction performance, which can be used for long-term prediction. Moreover, the stability of the prediction reflects our model can capture the apparent periodicity, such as daily periodicity, weekly periodicity, monthly periodicity.

### Validation of effectiveness

**Figure 11:**
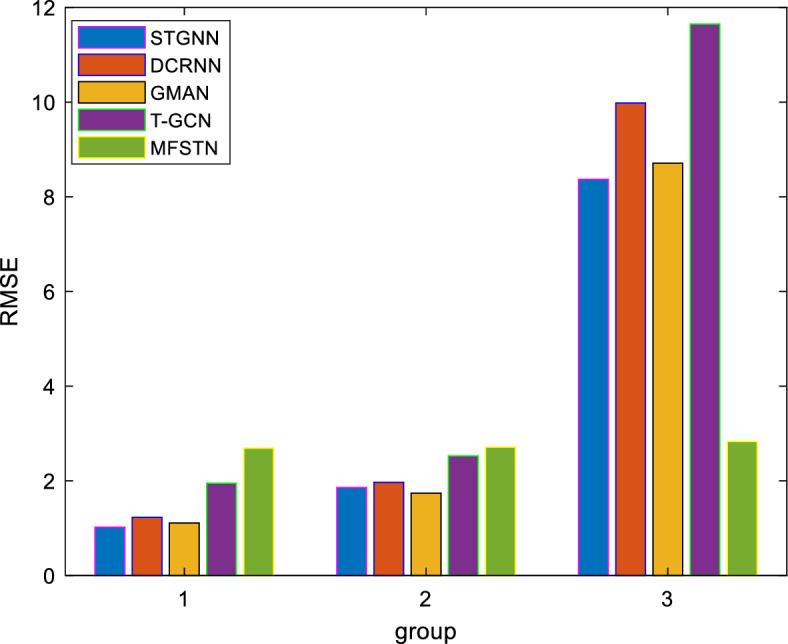
Validation of effectiveness of proposed model.

As we have already introduced, the most of state-of-the-art models are based on the continuous spatial–temporal sequences, which are not applicable to the scenarios with high data missing rate. Therefore, our model is not comparable to most of existing models. To show the effectiveness, we indirectly compare the proposed method with the advanced models. The details are as follows. Since the dataset includes over 6 million records for 132 roads, we classify roads into three groups based on different data missing rates. The low data missing rate is defined as a rate of less than 20%, the medium data missing rate is defined as 20% to 50%, and the high data missing is as a rate of more than 50%. Then, we compare the proposed method with STGNN^4^, DCRNN^5^, GMAN^6^ and T-GCN^18^ under different groups. As Fig. 11 shown, the first group represents low data missing rate, we can see that the state-of-the-art models achieve better results when the data quality is satisfactory. The second group represents medium data missing rate, we can see that the gap with baseline is reduced. The third group represents high data missing rate, we can see that the proposed model realizes best result when data missing rate is high. We can conclude that the proposed model is beneficial to improve the feasibility when the data missing rate is high.

### Adaptability across various scenarios

To verify the model’s robustness and adaptability across various scenarios, we conduct experiments on the METR-LA dataset. The METR-LA dataset records traffic speed statistics by loop detectors from 207 sensors on Los Angeles County freeways for four months, from March 1, 2012, to June 30, 2012. The prediction results are as Table 7.

**Table 7:** The prediction comparison on METR-LA dataset.

Method	MAE	MSE	RMSE	R2	MAPE
HA	3.5135	51.2844	7.1613	0.7224	10.0030
SVR	3.2071	44.2320	6.6507	0.7606	8.6664
FCN	3.1571	43.4020	6.5878	0.7651	8.5536
Transformer	3.1451	42.9080	6.5504	0.7677	8.6823
LSTM-GAT	3.1386	42.3550	6.5080	0.7707	8.6811
MFSTN	**3.1164**	**41.0470**	**6.4068**	**0.7768**	**8.6001**

Significant values are in bold.

It is worth mentioning that the METR-LA is a public dataset, whose average data missing rate is only 8.109%. The better data continuity allows us to add some adjacent time slices as supplementary features. However, this dataset does not provide the information of road shapes, such as the lengths, widths and areas. Although the dataset’s characteristics are different, the prediction results are still good. This demonstrates that our method can adapt to different dataset’s characteristics. As Table 7, we can see that the MFSTN is still the best predictor, followed by Transformer. The prediction of LSTM-GAT is better than before. This is because the data continuity is better, so that the recurrent network structure can play more advantages. Duo to the inherent limitations, the HA, SVR and FCN are still the bottom three. To sum up, the conclusions in the Table 7 are consistent with the Table 6. That shows that our method has the adaptability to various scenarios.

### Ablation study

To evaluate the effectiveness of each part in proposed method, we conduct ablation studies with 3 variants of our model on the real-world dataset from Guizhou as follows:

- w/o Medium-Level Features. It removes Medium-Level Features (MLF).
- w/o Low-Level Features. It removes Low-Level Features (LLF).
- w/o GAT. It removes Graph Attention Network (GAT).

**Figure 12:**
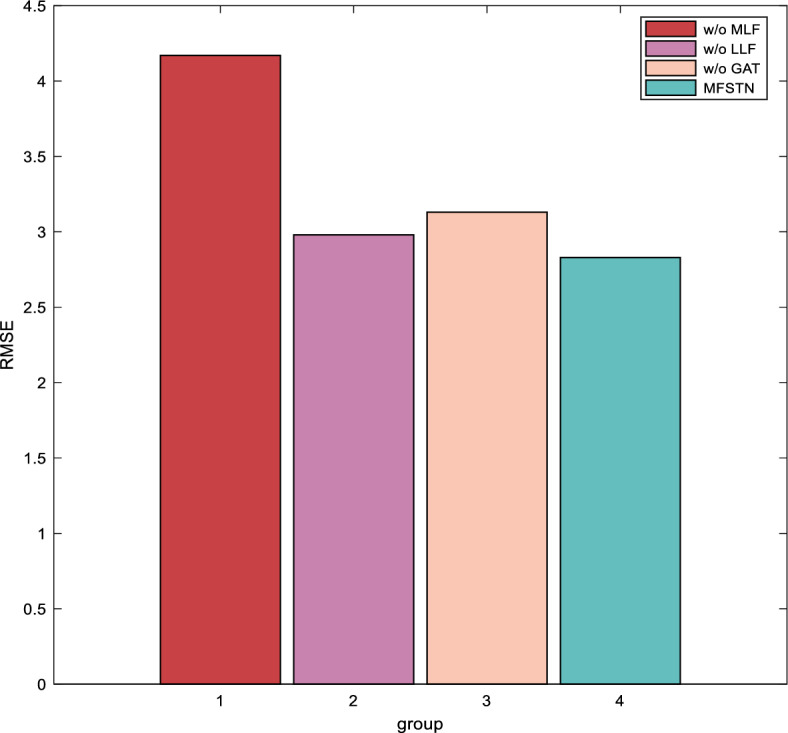
The model ablation.

As we introduced, we propose ATFEM and MFSTN in this paper. The ATFEM selects the high-level features, medium-level features and low-level features for the model. Then, the MFSTN processes the temporal features by TTE and spatial features by GAT. Since the high-level features play a decisive role in prediction, and the TTE is responsible for encoding high-level features, we don't take them into account in the ablation experiment. Then, we get the above 3 variants. As Fig. 12 shown, removing medium-level features will bring higher errors, followed by low-level features. It means that the higher the importance of the feature, the greater the effect. This shows the effectiveness of the proposed ATFEM. Moreover, the GAT is an important part of the model. This shows that GAT plays an important role in organizing spatial features. As Fig. 12 shown, removing the GAT will bring observable prediction errors. This shows that our model design is reasonable.

## Conclusion

In this paper, we extract multiple traffic features from the multi-source data. To improve the representative ability of the multiple traffic features, we propose the ATFEM, an Adaptive Traffic Features Extraction Mechanism, which selects significant features and construct the historical trends to get the JTFM and GSFM. To achieve the prediction based on the latent united representation of spatial–temporal features, we propose the MFSTN, a multi-feature spatial–temporal fusion network, which consists of TTE, GAT, our scaled spatial–temporal fusion module and MLP. In detail, the TTE is used to encode JTFM to get the joint temporal embedding, the GAT is used to aggregate GSFM to get the global spatial representation, our scaled spatial–temporal fusion module can automatically learn optimal fusion strategy to get the latent united representation of spatial–temporal features, the MLP outputs the prediction result. Experimental results show that the proposed model outperforms a variety of baselines. In the future, we will devote to the data quality improvement techniques and construct more powerful models to achieve more accurate results.

## Data Availability

All data included in this study are available upon request by contact with the corresponding author.

## References

[1] Tang F, Mao B, Kato N, Gui G (2021). Comprehensive survey on machine learning in vehicular network: Technology, applications and challenges. IEEE Commun. Surv. Tut..

[2] Zhao LP, Li F, Sun DY, Dai F (2023). Highway traffic crash risk prediction method considering temporal correlation characteristics. J. Adv. Transp..

[3] Guo, S., Lin, Y., Feng, N., Song, C. & Wan, H. Attention based spatial-temporal graph convolutional networks for traffic flow forecasting. In* Proceedings of the AAAI Conference on Artificial Intelligence* (2019).

[4] Yu, B., Yin, H. & Zhu, Z. Spatio-temporal graph convolutional networks: A deep learning framework for traffic forecasting. In* Proceedings of the International Conference on IJCAI* (2018).

[5] Li, Y., Yu, R., Shahabi, C. & Liu, Y. Diffusion convolutional recurrent neural network: Data-driven traffic forecasting. In* Proceedings of the International Conference on ICLR* (2018).

[6] Zheng, C., Fan, X., Wang, C. & Qi, J. GMAN: A graph multi-attention network for traffic prediction. In* Proceedings of the AAAI Conference on Artificial Intelligence* (2020).

[7] Liu J, Guan W (2004). A summary of traffic flow forecasting methods. J. Highway Transp. Res. Dev..

[8] Chen X, Sun L (2021). Bayesian temporal factorization for multidimensional time series prediction. IEEE T. Pattern. Anal..

[9] Qiu J, Jammalamadaka SR, Ning N (2018). Multivariate Bayesian structural time series model. J. Mach. Learn. Res..

[10] Williams BM, Hoel LA (2003). Modeling and forecasting vehicular traffic flow as a seasonal ARIMA process: Theoretical basis and empirical results. J. Transp. Eng..

[11] Hong WC (2011). Traffic flow forecasting by seasonal SVR with chaotic simulated annealing algorithm. Neurocomputing..

[12] Chen, M., Wei, Z., Huang, Z., Ding, B. & Li, Y. Simple and deep graph convolutional networks. In *International Conference on Machine Learning*, pp. 1725–1735 (2020).

[13] Zhang S, Liu YB, Xiao YP, He R (2023). Spatial-temporal upsampling graph convolutional network for daily long-term traffic speed prediction. J. King. Saud Univ. Com..

[14] Chen W, Li ZP, Liu C, Ai Y (2021). A deep learning model with Conv-LSTM networks for subway passenger congestion delay prediction. J. Adv. Transp..

[15] Guo G, Zhang T (2020). A residual spatio-temporal architecture for travel demand forecasting. Transp. Res C-Emer..

[16] Zhao F, Zeng GQ, Lu KD (2020). EnLSTM-WPEO: Short-term traffic flow prediction by ensemble LSTM, NNCT weight integration, and population extremal optimization. IEEE T. Veh. Technol..

[17] Song, C., Lin, Y., Guo, S. & Wan, H. Spatial-temporal synchronous graph convolutional networks: A new framework for spatial-temporal network data forecasting. In *Proceedings of the AAAI Conference on Artificial Intelligence,* pp. 914–921 (2020).

[18] Zhao L, Song Y, Zhang C, Liu Y, Wang P, Lin T, Deng M, Li H (2020). T-GCN: A temporal graph convolutional network for traffic prediction. IEEE Trans. Intell. Transp. Syst..

[19] Duan YX, Chen N, Shen SG, Zhang PY, Qu YY, Yu S (2022). FDSA-STG: Fully dynamic self-attention spatio-temporal graph networks for intelligent traffic flow prediction. IEEE T. Veh. Technol..

[20] He, H. T., Ye, K. J. & Xu, C. Z. Multi-feature urban traffic prediction based on unconstrained graph attention network. In *2021 IEEE International Conference on Big Data (Big Data)*, pp. 1409–1417 (2021).

[21] Shu WN, Cai K, Xiong NN (2021). A short-term traffic flow prediction model based on an improved gate recurrent unit neural network. IEEE Trans. Intell. Transp. Syst..

[22] Guo SN, Lin YF, Wan HY, Li XC, Cong G (2022). Learning dynamics and heterogeneity of spatial-temporal graph data for traffic forecasting. IEEE T. Knowl. Data En..

[23] Lou P, Wu ZH, Hu JW, Liu Q, Wei Q (2023). Attention-based gated recurrent graph convolutional network for short-term traffic flow forecasting. J. Math..

[24] Cirstea, R. G., Yang, B., Guo, C., Kieu, T. & Pan, S. Towards spatiotemporal aware traffic time series forecasting. In *2022 IEEE 38th International Conference on Data Engineering (ICDE)*, pp. 2900–2913 (2022).

[25] Wang Z, Su X, Ding Z (2020). Long-term traffic prediction based on lstm encoder-decoder architecture. IEEE T. Intell. Transp..

[26] Fang Y, Zhao F, Qin Y, Luo H, Wang C (2022). Learning all dynamics: Traffic forecasting via locality-aware spatio-temporal joint transformer. IEEE Trans. Intell. Transp. Syst..

[27] Reza S, Ferreira MC, Machado JJM, Tavares JMRS (2022). A multi-head attention-based transformer model for traffic flow forecasting with a comparative analysis to recurrent neural networks. Expert Syst. Appl..

[28] Huo GY, Zhang Y, Wang BY, Gao JB, Hu YL, Yin BC (2023). Hierarchical spatio-temporal graph convolutional networks and transformer network for traffic flow forecasting. IEEE Trans. Intell. Transp. Syst..

[29] Guo G, Yuan W (2020). Short-term traffic speed forecasting based on graph attention temporal convolutional networks. Neurocomputing.

[30] Huang J, Luo K, Cao LB, Wen YQ, Zhong SY (2022). Learning multiaspect traffic couplings by multirelational graph attention networks for traffic prediction. IEEE Trans. Intell. Transp. Syst..

